# Altered gut microbiota in individuals with episodic and chronic migraine

**DOI:** 10.1038/s41598-023-27586-4

**Published:** 2023-01-12

**Authors:** Dongeun Yong, Hakbae Lee, Hyung-Gyu Min, Kyungnam Kim, Hyun-Seok Oh, Min Kyung Chu

**Affiliations:** 1 Department of Laboratory Medicine and Research Institute of Bacterial Resistance, Yonsei University College of Medicine, Seoul, Republic of Korea; 2 Department of Statistics and Data Science, Yonsei University College of Commerce and Economics, Seoul, Republic of Korea; 3 CJ Bioscience Inc., Seoul, Republic of Korea; 4 Department of Neurology, Severance Hospital, Yonsei University College of Medicine, 50-1 Yonsei-Ro, Seodaemun-Gu, Seoul, 03722 Republic of Korea

**Keywords:** Ecology, Microbiology, Neurology

## Abstract

Emerging evidence reveals a close association between gut microbiota and human neurological disorders. The present study aimed to assess whether the composition of gut microbiota in participants with episodic migraine (EM) and chronic migraine (CM) was altered in comparison to that of the controls. This study was a cross-sectional, case–control study. The gut microbiota were evaluated by the partial, targeted sequencing of the 16S rRNA V3–V4 region. This study enrolled 42 and 45 participants with EM and CM, respectively, and 43 controls. Alpha and beta diversities revealed no significant difference among the three groups; however, the microbiota composition at the class, order, family, and genus levels differed significantly between EM and the control, CM and the control, and the EM and CM groups. Moreover, higher composition of *PAC000195_g* was significantly associated with a lower headache frequency among the five genera that exhibited significantly different microbiota composition in EM and CM. *Agathobacter* revealed a significant negative association with severe headache intensity. The findings of the present study provide evidence of altered gut microbiota in EM and CM. These findings will help in understanding the course and treatment of migraine.

## Introduction

The term “gut-brain axis” indicates the bidirectional relationship between the gastrointestinal (GI) system and the brain^1^, that is, the brain regulates the function of GI system, and the GI system has profound effects on the brain. The “gut-brain axis” is influenced by several factors, such as inflammatory mediators, neuropeptides, dietary intakes, metabolites, and gut microbiota profiles^2^. Moreover, microbiota play a pivotal role in the gut-brain axis^3^, wherein they affects nociception via immune, neural, endocrine, and metabolic signaling^4^.

Altered gut microbiota composition has been reported in metabolic, cardiovascular, oncologic, neurologic, and psychiatric disorders^5^. Emerging evidence suggests that gut microbiota are crucial in the pathogenesis of pain disorders including headache^3,6^.

A close association has been identified between migraine and GI disturbances, that is, individuals with migraine exhibit a higher prevalence of GI disturbances including diarrhea, constipation, dyspepsia, and gastroesophageal reflux compared to those without migraine^7^. Furthermore, altered gut microbiota has been observed in GI disorders such as functional dyspepsia, irritable bowel syndrome, and gastroesophageal reflux. Nevertheless, information on the microbiota composition in individuals with migraine is currently limited^7,8^.

Based on these findings on the association of microbiota, GI disturbances, and migraine, we hypothesized that participants with migraine presented altered composition of gut microbiota compared with the controls. The present study aimed (1) to evaluate whether the composition of gut microbiota in participants with episodic migraine (EM) and chronic migraine (CM) (common subtypes of migraine) was altered in comparison to that in the controls and (2) to assess the association of microbial composition with clinical characteristics and comorbidities of migraine.

## Methods

### Participants and clinical evaluation

Participants with EM and CM were recruited from the neurology out-patient clinic of a tertiary-care university hospital between February, 2020 and November, 2020. The inclusion criteria for participants with EM and CM were: (1) age 19–65 years and (2) those who fulfilled the third edition of the International Classification of Headache Disorders (ICHD-3) criteria of EM (code 1.1 or 1.2) or CM (code 1.3)^9^. The exclusion criteria were: (1) current medical or psychiatric treatment other than for anxiety, depression, and fibromyalgia; (2) remarkable dietary habit changes that occurred within six months prior to the study; and (3) history of taking probiotics or antibiotics in the previous year. The diagnosis of EM and CM, evaluation of clinical characteristics, and comorbidities of participants were conducted through a doctor’s (MKC) interview based on diagnostic criteria. Controls were recruited via advertisement after matching for age, sex, and body mass index (BMI) distributions in the EM and CM groups. Controls were eligible if they had not experienced headaches during the previous year and if they had not reported migraine or probable migraine attacks during their lifetime. Participants with diarrhea were not included. Individuals consuming probiotics were excluded from the control group. All participants in the EM, CM, and control groups were not related or living together.

### Assessment of anxiety, depression, and fibromyalgia

Since anxiety, depression, and fibromyalgia (FM) are common comorbidities of migraine, we assessed their association with microbiota change in participants presenting migraine. Anxiety and depression were evaluated using Generalized Anxiety Disorder-7 (GAD-7) and Patient Health Questionnaire-9 (PHQ-9), respectively. If GAD-7 score was ≥ 8 and PHQ score was ≥ 10, the participant was classified as having anxiety and depression, respectively. GAD-7 and PHQ-9 were previously validated in Korean language^10,11^. Fibromyalgia was diagnosed according to the 2016 American College of Rheumatology criteria^12,13^.

### Sample acquisition and handling

Participants were advised to collect a fecal sample at home during the initial interview. The fecal sample was collected using a fecal sample collection kit (SPL Korea, Seoul, Korea). Thereafter, the samples were fresh-frozen at − 20 ℃, and were delivered by the participants to the study site within 14 days of collection. On arrival at the study site, the fecal samples were stored at − 70 ℃ before further analysis.

### DNA extraction

DNA extraction was performed using the FastDNA Spin Kit for Soil (MP Biomedicals, Irvine, California, USA), according to manufacturer’s instructions. In general, 10 µL of fecal sample was extracted from the sample of each participant and released in 978 µL of phosphate buffer and 122 µL of MTP™ (Sigma Aldrich, Burlington, VT, USA) buffer. Thereafter, they were vortexed vigorously until the fecal samples were thoroughly homogenized. The extraction process was performed according to the kit protocols. DNA was quantified by measuring absorbance at 260 and 280 nm using a NanoDrop ND-1000 spectrophotometer (NanoDrop Technology, Rockland, DE, USA) and Quantus™ Fluorometer (Promega, Madison, WI, USA).

### PCR and partial targeted sequencing of the 16S rRNA V3–V4 region

PCR was performed to amplify the template in the DNA samples using V3–V4 region primers with overhang adapters attached, which included 16S_V3_F (5′-TCG TCG GCA GCG TCA GAT GTG TAT AAG AGA CAG CCT ACG GGN GGC WGC AG-3′) and 16S_V4_R (5′-GTC TCG TGG GCT CGG AGA TGT GTA TAA GAG ACA GGA CTA CHV GGG TAT CTA ATC C-3′). Amplification was carried out in two steps under the following conditions: first step of initial denaturation at 95 °C for 3 min, followed by 25 cycles of 98 °C for 30 s, 55 °C for 30 s, and 72 °C for 30 s, with a final elongation at 72 °C for 5 min; the second step of initial denaturation was at 95 °C for 3 min, followed by 8 cycles of 98 °C for 30 s, 60 °C for 30 s, and 72 °C for 30 s, with a final elongation at 72 °C for 5 min. The PCR product was confirmed by 1% agarose gel electrophoresis and visualized under a G: BoxiChemi XL system (Syngene, Cambridge, UK). After attaching Nextera^®^ XT Index Kit V2, an Illumina adapter primer, sequencing was performed using an Illumina V3 600 cycle cartridge and Illumina MiSeq equipment (San Diego, California, USA).

### Sequencing data analyses

The obtained sequence reads were analyzed using Chunlab program (Chunlab, Inc., Seoul, Korea) and operational taxonomic unit (OTU)-based analyses. For initial quality filtering and rarefaction analysis, we selected only observations with at least 10,000 reads in the dataset. The taxonomic classification of each read was assigned against the EzBioCloud^14^, a united database of 16S rRNA gene sequences; whole-genome assemblies were used for taxonomic assignment using BLAST 2.2.22, and pairwise alignments were generated to calculate similarity^15^. We used the same methodology as that employed in a previous study^16^. To calculate the relative abundance of gut microbiota at different levels, the summaries of taxonomic distributions of OTUs were constructed using these taxonomies. Three parameters (Chao1, Shannon, and Simpson indices) were used to evaluate alpha diversity at a 3% distance^17,18^. Beta diversity was assessed in the principal coordinate analysis (PCoA) plot with the weighted UniFrac distance, the unweighted UniFrac distance, and the Bray–Curtis dissimilarity index^19,20^. Since partial targeted sequencing of the 16S rRNA V3–V4 region may not provide enough coverage of variable regions to allow unambiguous species-level identification of numerous important human bacterial microbiota, we did not evaluate the microbiota alteration at the species level in the present study^21^.

### Statistical analysis

Statistical analyses were performed using EzBioCloud 16S-based Microbiome Taxonomic Profiling, which is a ChunLab’s bioinformatics cloud platform^22^. The Wilcoxon rank-sum test was used to test the difference between groups in the number of OTU and relative abundance of specific taxa. A two-tailed *p*-value < 0.05 and false discovery rate-adjusted *p*-value < 0.1 were considered significant. Taxonomic biomarkers were discovered by statistical comparison algorithms using the linear discriminant analysis effect size method^23^.

We performed additional analyses for the association of microbiota with clinical characteristics and comorbidities of migraine at the genus level. We used Poisson regression analyses for the association of headache frequency and relative abundance of microbiota at the genus level, adjusting for age, sex, and BMI. For the association of severe headache intensity, anxiety, depression, and fibromyalgia with the relative abundance of microbiota, we used logistic regression analyses (adjusting for age, sex, and BMI) using the R package, *vegan*, version 2.5–6^24^. No statistical power calculation was conducted before commencing the study, and the sample size used was based on available data. Nevertheless, the sample size of the present study was not smaller than that of similar, previous studies on microbiota characterization in other neurological conditions^25^. There were no missing data in our study.

### Ethical considerations

This study was approved by the institutional review board/ethics committee of Severance Hospital, Yonsei University (IRB No. 2019-3509-006). Written informed consent was obtained from all participants before inclusion in the study. All methods were carried out in accordance with the relevant guidelines and regulations.

## Results

### Participants

**Figure 1:**
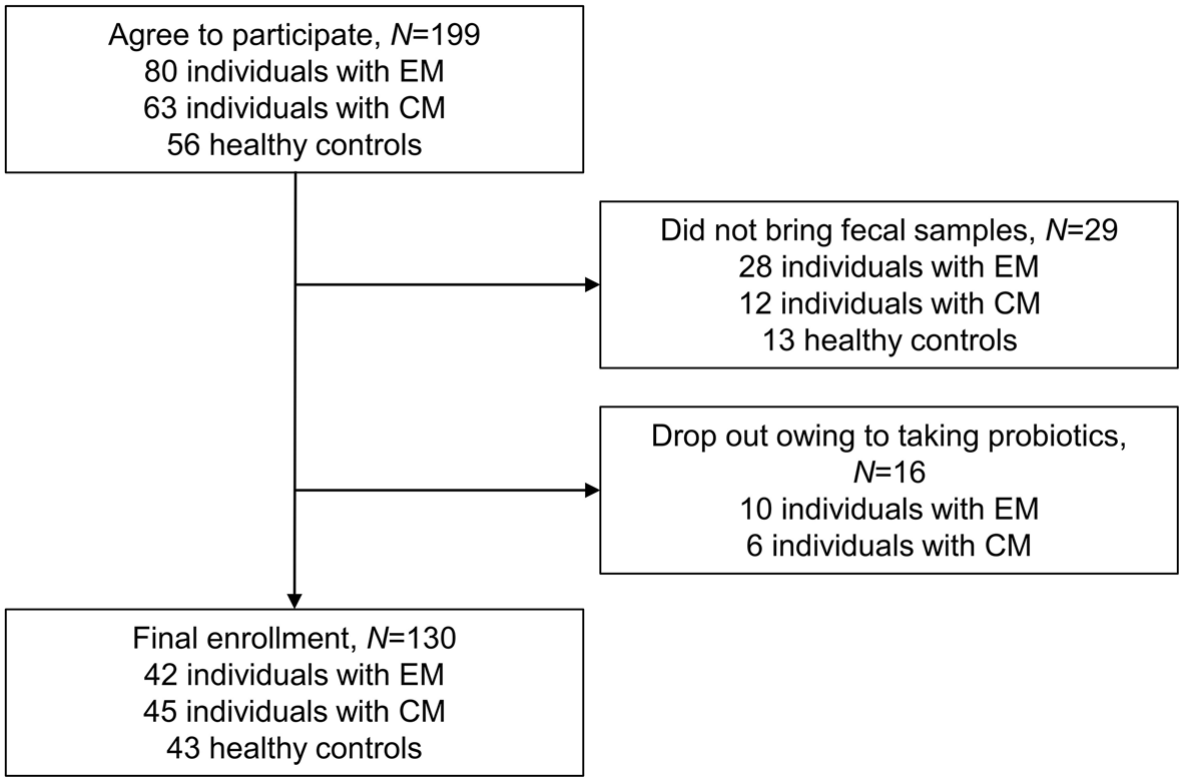
Flow of participants in a study on the composition of gut microbiota in participants with episodic or chronic migraine.

In total, 80, 63, and 56 participants in the EM, CM, and control groups, respectively, initially agreed to participate in this study. Nevertheless, 28, 12, and 13 individuals in the EM, CM, and control groups, respectively, withdrew their participation and did not bring any fecal samples to the study site. After providing fecal samples, 10 and 6 individuals with EM and CM, respectively, reported intake of probiotics and were excluded from the analysis. No participant in the control group consumed probiotics during the study period. Eventually, 42, 45, and 43 participants in the EM, CM, and control groups, respectively, were enrolled (Fig. 1). The demographic and clinical characteristics of participants are summarized in Table 1. All participants with EM and CM used acute treatments for migraine. Moreover, 25 (59.5%) and 27 (60.0%) participants with EM and CM, respectively, received prophylactic treatment for migraine. Of the 42 participants with EM, 20 used anti-epileptic medications, 11 used beta blockers, 2 used an anti-depressant, and 1 used a calcium-channel blocker for prophylactic treatment. Of the 45 participants with CM, 23 used anti-epileptic medications, 8 used beta blockers, 1 used an anti-depressant, and no participant used calcium-channel blockers for prophylactic treatment. No participant in the EM, CM, and control groups was infected with SARS-CoV-2 before or during participation in the study.

**Table 1:** Demographic and clinical characteristics of participants with episodic and chronic migraine and the control.

	Episodic migraine, *N* = 42	Chronic migraine, *N* = 45	Controls, *N* = 43	*P*-value
Age	39.6 ± 11.4	40.8 ± 12.5	43.2 ± 11.7	0.350*
Women, *N* (%)	33 (78.6)	41 (91.1)	35 (81.4)	0.246*
Body Mass Index	22.8 ± 2.5	22.7 ± 3.5	22.1 ± 3.6	0.591*
Headache frequency per month	6.8 ± 5.4	20.7 ± 7.5		< 0.001^†^
Severe headache intensity	33 (78.6)	39 (86.7)		0.318^†^
Unilateral pain, N (%)	28 (66.7)	19 (42.2)		0.022^†^
Pulsating quality, *N* (%)	41 (97.6)	44 (97.8)		1.000^†^
Aggravation by movement, *N* (%)	35 (83.3)	43 (95.6)		0.083^†^
Nausea, *N* (%)	40 (95.2)	42 (93.3)		1.000^†^
Vomiting, *N* (%)	10 (23.8)	16 (35.6)		0.232^†^
Photophobia, *N* (%)	15 (35.7)	23 (51.1)		0.148^†^
Phonophobia, *N* (%)	21 (50.0)	25 (55.6)		0.604^†^
Impact of headache (Headache Impact Test-6 score)	59.0 ± 8.1	61.7 ± 7.4		0.280^†^
Anxiety (GAD-7 score ≥ 8), *N* (%)	12 (28.6)	16 (35.6)		0.486^†^
Depression (PHQ-9 score ≥ 10), *N* (%)	11 (26.2)	20 (44.4)		0.076^†^
Fibromyalgia, *N* (%)	12 (28.6)	22 (48.9)		0.052^†^
Preventive medications, *N* (%)	25 (59.5)	27 (60.0)		0.869^†^

*EM* episodic migraine, *CM* chronic migraine.

*Compared among participants in the EM, CM, and control groups.

^†^Compared between participants with EM and CM.

### Collection of 16 s RNA sequencing data

We obtained 7,802,425 read sequences, accounting for 99.8% of the valid sequences from the fecal samples of 130 participants. According to barcode and primer sequence filtering, an average of 59,305 (range, 3716–90,832) observed sequences per sample was recovered for downstream analysis. Thus, 2,242,325 sequences were obtained from the controls for phylogenetic analysis, whereas 2,747,952 and 2,812,148 sequences were obtained from the EM and CM groups, respectively.

### Microbial diversity

**Figure 2:**
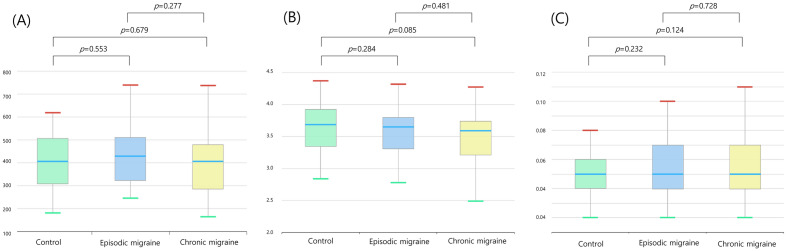
Alpha diversity at the genus level using Chao1 (**A**), Shannon (**B**), and Simpson (**C**) indices^*,†^. ^*^Controls (green) and participants with episodic migraine (blue) and chronic migraine (yellow). ^†^In the box plots, the lower boundary of the box indicates the 25th percentile; a blue line within the box marks the median, and the upper boundary of the box indicates the 75th percentile. Whiskers above (red) and below the box (green) indicate the highest and the lowest values, respectively.

**Figure 3:**
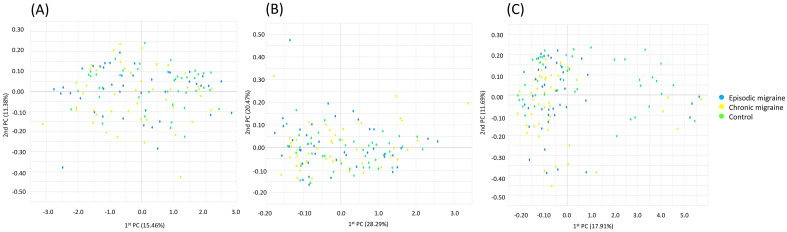
Beta diversity of microbiota in principal coordinate analysis plot with the weighted UniFrac distance (**A**), the unweighted UniFrac distance (**B**) and the Bray–Curtis dissimilarity index (**C**)^*^. ^*^Controls (green) and participants with episodic migraine (blue) and chronic migraine (yellow).

Alpha diversity was defined as microbial community richness and evenness. Alpha diversities in the genus richness, as evaluated by Chao1 (Fig. 2A), Shannon (Fig. 2B), and Simpson (Fig. 2C) indices, did not differ significantly among the EM, CM, and control groups. Beta diversity represented the community composition dissimilarity between samples. PCoA with the weighted UniFrac distance (Fig. 3A and Supplementary Fig. S1A, *p* = 0.176, permutational multivariate analysis of variance [PERMANOVA]), the unweighted UniFrac distance (Fig. 3B and Supplementary Fig. S1B, *p* = 0.132, PERMANOVA), and the Bray–Curtis dissimilarity index (Fig. 3C and Supplementary Fig. S1C, *p* = 0.220, PERMANOVA) for beta diversity at the genus level among the EM, CM, and control groups revealed that these three groups could not be separated.

### Relative abundance of fecal microbes between participants with EM and the control

**Figure 4:**
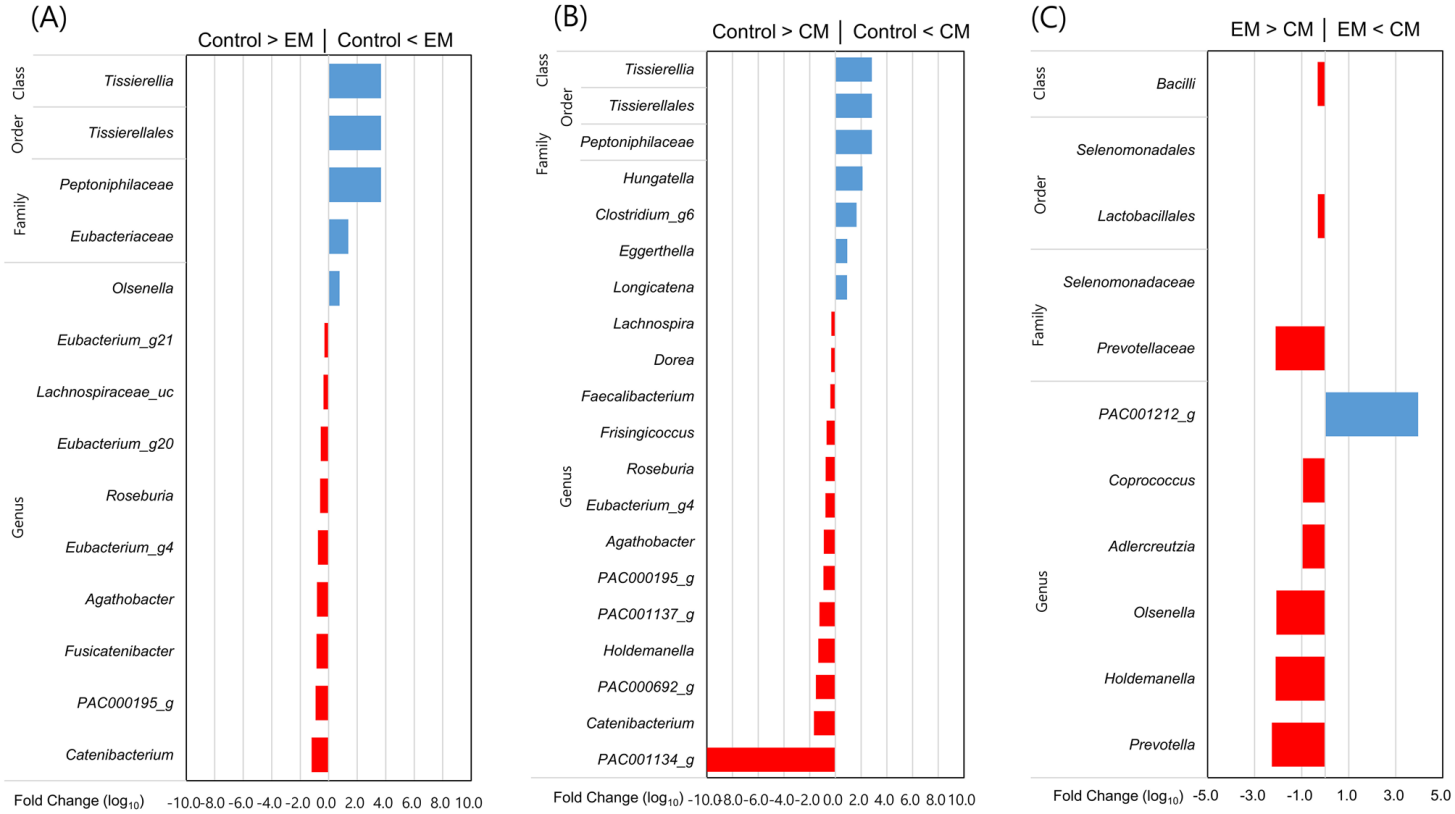
Taxonomic differences in fecal microbiota among participants. The fold change (log_2_) denotes the difference in relative abundance between participants with episodic migraine and the control (**A**), between those with chronic migraine and the control (**B**), and between those with episodic and chronic migraine (**C**). *CM* chronic migraine; *EM* episodic migraine.

Relative abundance of fecal microbes at the phylum level did not differ significantly among participants in the control, EM, and CM groups (Supplementary Fig. S2). Moreover, *Tissierellales* (*p* = 0.001) and *Tissierellia* (*p* = 0.001) were more abundant in the EM group than that in the control group at the order and class levels, respectively (Fig. 4A). At the family level, *Peptoniphilaceae* (*p* = 0.001) and *Eubacteriaceae* (*p* = 0.045) occurred at a significantly higher proportion in the EM group than that in the control group. Furthermore, at the genus level, the abundance of 11 genera differed significantly between the two groups, including one more abundant and 10 less abundant genera in the EM group. *Catenibacterium* (*p* = 0.031) and *Olsenella* (*p* = 0.038) had the highest relative abundance in the control and EM groups, respectively.

### Relative abundance of fecal microbes between participants with CM and the control

The analysis results at the class, order, family, genus, and species levels between CM and control groups are illustrated in Fig. 4B. *Tissierellia* (*p* = 0.001)*, Tissierellales* (*p* = 0.001)*,* and *Peptoniphilaceae* (*p* = 0.001) were more abundant in the CM group than that in the control group at the class, order, and family levels, respectively; however, at the genus level, the abundances of 18 genera differed significantly, including four more abundant and 14 less abundant genera in the CM group than in the control group.

### Relative abundance of fecal microbes between participants with EM and CM

The analysis results at the class, order, family, and genus levels between CM and EM groups are summarized in Fig. 4C. At the class level, *Bacilli* (*p* = 0.033) were less abundant in the CM group than that in the EM group; however, at the order level, *Selenomonadales* (*p* = 0.016) and *Lactobacillales* (*p* = 0.034) were less abundant in the CM group than that in the EM group. Moreover, at the class level, *Selenomonadaceae* (*p* = 0.016) and *Prevotellaceae* (*p* = 0.012) were less abundant in the CM group than that in the EM group. Furthermore, at the genus level, *PAC001212_g* (*p* = 0.019) revealed relative positive predominancy in the CM groups, whereas *Prevotella* (*p* = 0.019)*, Holdemanella* (*p* = 0.009)*, Olsenella* (*p* = 0.033)*, Adlercreutzia* (*p* = 0.018)*,* and *Coprococcus* (*p* = 0.040) revealed relative positive predominancy in the EM group.

### Association among fecal microbiota and clinical characteristics and comorbidities of migraine

Among the five genera (*Roseburia, Eubacterium_g4, Agathobacter, PAC000195_g,* and *Catenibacterium*) depicting predominance or less-predominance both in EM and CM groups, we conducted additional analyses for clinical characteristics and migraine comorbidities.

Combining the results of the 42 and 45 participants with EM and CM, respectively, the Poisson regression analysis for relative abundance of microbiota revealed that a higher composition of *PAC000195_g* (*p* = 0.040) was significantly associated with lower headache frequency (Table 2). Furthermore, *Agathobacter* (*p* = 0.009) had a negative association with severe headache intensity (Table 3). Anxiety was associated with *Catenibacterium* (*p* = 0.027); however, depression did not reveal any association with the five genera (Table 3)*.*

**Table 2:** The association between headache frequency and the relative abundance of microbiota.^*^

Genus	Beta	Standard error	*P*-value
*Roseburia*	− 0.189	0.126	0.125
*Eubacterium_g4*	− 0.152	0.468	0.744
*Agathobacter*	− 0.076	0.050	0.128
*PAC000195_g*	− 0.509	0.248	0.040
*Catenibacterium*	− 0.007	0.013	0.050

*Poisson regression analyses at the genus level. Changes in headache frequency per month according to 1% increase in the composition of microbiota.

**Table 3:** The association of severe headache intensity and comorbidities with the relative abundance of microbiota*.

Variables	Odds ratio and 95% confidence interval	*P*-value
Severe headache intensity		
* Roseburia*	0.829 (0.551–1.246)	0.367
* Eubacterium_g4*	0.428 (0.082–2.243)	0.315
* Agathobacter*	0.789 (0.660–0.942)	0.009
* PAC000195_g*	0.583 (0.266–1.278)	0.178
* Catenibacterium*	0.906 (0.811–1.012)	0.080
Fibromyalgia		
* Roseburia*	0.872 (0.520–1.461)	0.602
* Eubacterium_g4*	0.067 (0.004–1.036)	0.053
* Agathobacter*	0.954 (0.785–1.159)	0.633
* PAC000195_g*	0.529 (0.202–1.383)	0.194
* Catenibacterium*	0.679 (0.458–1.008)	0.055
Anxiety (GAD-7 ≥ 8)		
* Roseburia*	0.654 (0.411–1.043)	0.075
* Eubacterium_g4*	0.217 (0.030–1.561)	0.129
* Agathobacter*	0.999 (0.845–1.183)	0.997
* PAC000195_g*	0.757 (0.335–1.709)	0.502
* Catenibacterium*	0.836 (0.713–0.979)	0.027
Depression (PHQ-9 ≥ 10)		
* Roseburia*	0.726 (0.374–1.409)	0.344
* Eubacterium_g4*	0.110 (0.005–2.367)	0.159
* Agathobacter*	1.206 (0.999–1.471)	0.063
* PAC000195_g*	0.368 (0.111–1.216)	0.101
* Catenibacterium*	0.725 (0.520–1.011)	0.059

*GAD-7* Generalized Anxiety Disorder-7; *PHQ-9* Patient Health Questionnaire-9.

*Logistic regression analyses at the genus level.

### Relative abundance of fecal microbes in participants with EM based on prophylactic treatment

Alpha and beta diversities in participants with EM did not differ significantly based on their prophylactic treatment (Supplementary Figs S3A–C, S4A–C, and S5A–C). At the genus level, *Klebsiella* (*p* = 0.009)*, Enterobacteriaceae_g* (*p* = 0.006)*,* and *Faecalibacterium* (*p* = 0.046) were more abundant in the prophylactic group than the non-prophylactic group (Supplementary Fig. S6A).

### Relative abundance of fecal microbes in participants with CM based on prophylactic treatment

Alpha and beta diversities in participants with CM did not differ significantly based on prophylactic treatment (Supplementary Figs S7A–C, S8A–C, and S9A–C). *Emergencia* (*p* = 0.043)*, Ruthenibacterium* (*p* = 0.005)*, Eggerthella* (*p* = 0.003)*, PAC000743_g* (*p* = 0.034)*,* and *Anaerostipes* (*p* = 0.039) were more abundant in the prophylactic group, whereas *PAC000196_g* (*p* = 0.049)*, Fusicatenibacter* (*p* = 0.028)*,* and *Faecalibacterium* (*p* = 0.021) were more abundant in the non-prophylactic group at the genus level (Supplementary Fig. S6B).

## Discussion

The main findings of the present study were as follows: (1) no significant difference was observed in the alpha and beta diversities of microbiota among the participants in EM, CM, and control groups; (2) significant difference in gut microbiota at the class, order, family, and genus levels were observed between EM and control group, CM and control group, and EM and CM group; and (3) some clinical characteristics and comorbidities revealed significant association with the relative abundance of microbiota at the genus level.

Numerous studies have reported considerable alteration of microbiota in human pain disorders as well as in preclinical studies^6^. FM is the most common form of chronic widespread pain; individuals with FM have altered composition of microbiota at the genus and species levels compared with healthy controls^26^. Moreover, considerable alteration of gut microbiota is observed in individuals with irritable bowel syndrome, a common gastrointestinal pain disorder^27^. Furthermore, several studies report the effect of probiotics in migraine treatment. Probiotics supplements improved the quality of life in 40 individuals with migraine in an open label study. Nevertheless, these studies did not evaluate the change in headache frequency and intensity^28^. Another open-label study on probiotic supplement reported a significant decrease in the number of migraine days and disability after 12-week treatment^29,30^. In contrast, no significant difference was observed in the reduction of migraine duration between placebo and treatment groups after 12-week probiotic treatment in a randomized control study^30^. A recent study of elderly female twins reported altered gut microbiota in individuals with migraine^31^. However, the study did not describe clinical characteristics of migraine and did not distinguish between EM and CM. Additionally, the study population was limited to female twins showing gut microbiota heritability and as the prevalence of migraine remarkably decreases in elderly population, application of these results for general population is limited^31,32^.

Migraine can be divided into CM and EM. CM is a chronic form of migraine, which was defined as having headache for ≥ 15 days per month with ≥ 8 migraine features for > 3 months^9^. CM is less prevalent, has more comorbidities, presents higher cutaneous allodynia, and is less responsive to treatment than EM^33^. Therefore, we separately analyzed microbiota alteration in CM and EM and compared it to that of the controls.

The present study found that *Tissierellia, Tissierellales,* and *Peptoniphilaceae* were more abundant in EM and CM at the class, order, and family levels compared to that of the controls. Moreover, at the genus level, *Roseburia, Eubactgerium_g4, Agathobacter, PAC000195_g,* and *Catenibacterium* were more abundant both in EM and CM. Nevertheless, *Eubacteriacea*e at the family level was more abundant only in EM but not in CM. Other altered genera in EM did not reveal significant change in CM and vice versa (Fig. 4A–B). Furthermore, the microbiota in EM and CM revealed different relative abundance at the class, order, family, and genus levels (Fig. 4C). The results indicated that CM and EM revealed similar features in microbiota alteration at the class, order, family, and genus levels; however, few differences were also observed.

We observed a quantitative association between the relative abundance of several genera and headache frequency. Headache frequency is a key parameter of migraine severity. Moreover, it is closely related with more disability and effect of headache, more central sensitization, and comorbidities including insomnia, anxiety, and depression^34–36^. The quantitative association between headache frequency and relative abundance of several genera, which were altered in EM and CM, allowed us to confirm the alteration in microbiota in individuals with migraine.

We found that the relative abundance of *Catenibacterium* was significantly altered according to the presence of anxiety among the four genera that showed significant change in the EM and CM groups compared to that of the control. Nevertheless, we did not find significant change in the four genera with respect to the presence of depression. Considering that altered gut microbiota are repeatedly found in individuals with anxiety and depression, there is a possibility of a significant change in gut microbiota outside the four genera identified in participants with migraine^37,38^. The present study evaluated microbiota change according to the presence of anxiety, depression, and FM since they are prevalent among individuals with migraine, and symptoms of these disorders are more severe with the increase in the severity of migraine^34,39^. Therefore, we focused on the four genera and assessed microbiota change according to the presence of anxiety, depression, and FM. As comorbidities are important for the diagnosis, treatment, and understanding of the pathological mechanisms of migraine, further microbiota studies in individuals with migraine with respect to various comorbidities are required.

Additionally, in the present study, we observed that some microbiota at the genus level revealed significantly different relative abundance based on prophylactic treatment (Supplementary Fig. S4A–B). Except for *Faecalibacterium* and *Eggerthella* in CM, the altered microbiota based on prophylactic treatment did not overlap with the altered microbiota in EM and CM (Fig. 4A–B). These findings indicate that the five genera (*Roseburia, Eubacterium_g4, Agathobacter, PAC000195_g,* and *Catenibacterium*) that revealed altered relative abundance in EM and CM may not be severely affected by prophylactic treatment.

We found significant differences in the relative abundance of fecal microbiota according to the use of prophylactic medications in participants with EM and CM. This finding suggests that oral prophylactic medications can affect the gut microbiota of participants with EM and CM. Therefore, the effect of prophylactic medications should be considered when performing gut microbiota evaluation. Our findings also propose the possibility that prophylactic medications exert their effects by alternating gut microbiota. Further longitudinal studies will provide more information on the association between prophylactic medications and gut microbiota in individuals with EM and CM.

The present study has certain limitations. First, we did not evaluate the dietary intake of participants. Gut microbiota are affected by intrinsic and extrinsic factors and dietary intake is an important extrinsic factor. The effect of dietary intake on the gut microbiome has been consistently reported^40^. Recent studies have reported that dietary intervention may influence the disease status^41^. Nevertheless, we excluded individuals who changed their dietary habits within six months prior to the study and consumed probiotics. Although full dietary intake evaluation is difficult to conduct and has been performed only in few gut microbiota studies, further studies on microbiota with dietary assessment will be informative^26^. Second, we did not assess neurotrophic factors and metabolites. It has been hypothesized that epigenetic regulations by microbiota are mediated through neurotrophic factors and bioactive metabolites^42^. Brain-derived neurotrophic factor is involved in neurodevelopment via the microbiota–gut interaction^43^. Bioactive metabolites such as short chain fatty acids are involved in microbiota–host chemical communication^44^. Assessment of neurotrophic factors and bioactive metabolites together with the microbiota may provide better insight into the microbial effect on disease. Third, the present study cross-sectionally investigated the association of altered microbiota among participants in the EM, CM, and control groups. Therefore, we could not identify a cause–effect relationship in the present study. If the causal relationship between gut microbiota alteration in EM and CM is established, it would pave way for developing new treatment of EM and CM targeting gut microorganisms. Fourth, we did not check the stool state of participants. The stool state can be associated with gut microbiota^45,46^. Nevertheless, we only included participants without diarrhea in the present study. Stool state can be evaluated using instruments such as the Bristol Stool Scale (BSS)^47^. The BSS presents images which illustrate feces with precise descriptions, and classifies the forms of human feces into seven categories. The BSS has demonstrated its utility in clinical and experimental fields^48,49^. Further studies on the gut microbiota, including the state of feces using validated instruments, will provide more valuable information.

The strengths of the present study were as follows. First, we found a significant association between microbiota composition changes and clinical characteristics of migraine, including headache frequency and severe headache intensity. This finding may provide additional evidence of the alteration of gut microbiota in participants with migraine in addition to difference in the microbiota composition of EM and CM groups compared to the control. Second, the present study enrolled well-characterized participants with EM and CM comparable to control participants, matched with age and sex from the same region for the same period. With this setting, we could accurately identify the gut microbiota change in the EM and CM groups relative to the control.

## Conclusions

In conclusion, altered microbiota at multiple levels of taxa were identified in participants with EM and CM compared to that of the controls. Moreover, differences in microbiota composition between EM and CM were also observed. Some altered microbiota at the genus level revealed a relationship with clinical characteristics and comorbidities of migraine. Our findings may provide evidence for the altered gut microbiota in EM and CM. Furthermore, longitudinal studies would allow us to have more information on the relationship of microbiota with EM and CM and the effect of prophylactic treatment on microbiota in migraine.

## Supplementary Information

Supplementary Information 1.

Supplementary Information 2.

Supplementary Information 3.

Supplementary Information 4.

Supplementary Information 5.

Supplementary Information 6.

Supplementary Information 7.

Supplementary Information 8.

Supplementary Information 9.

Supplementary Information.

## Data Availability

The datasets generated and analyzed during the current study are available at https://doi.org/10.6084/m9.figshare.19310930.v1.
