# Supplementary figures and images for: Altered gut microbiota in individuals with episodic and chronic migraine
Source: Sci Rep. 2023 Jan 12;13:626. doi: 10.1038/s41598-023-27586-4 (PMC9835027; doi:10.1038/s41598-023-27586-4)

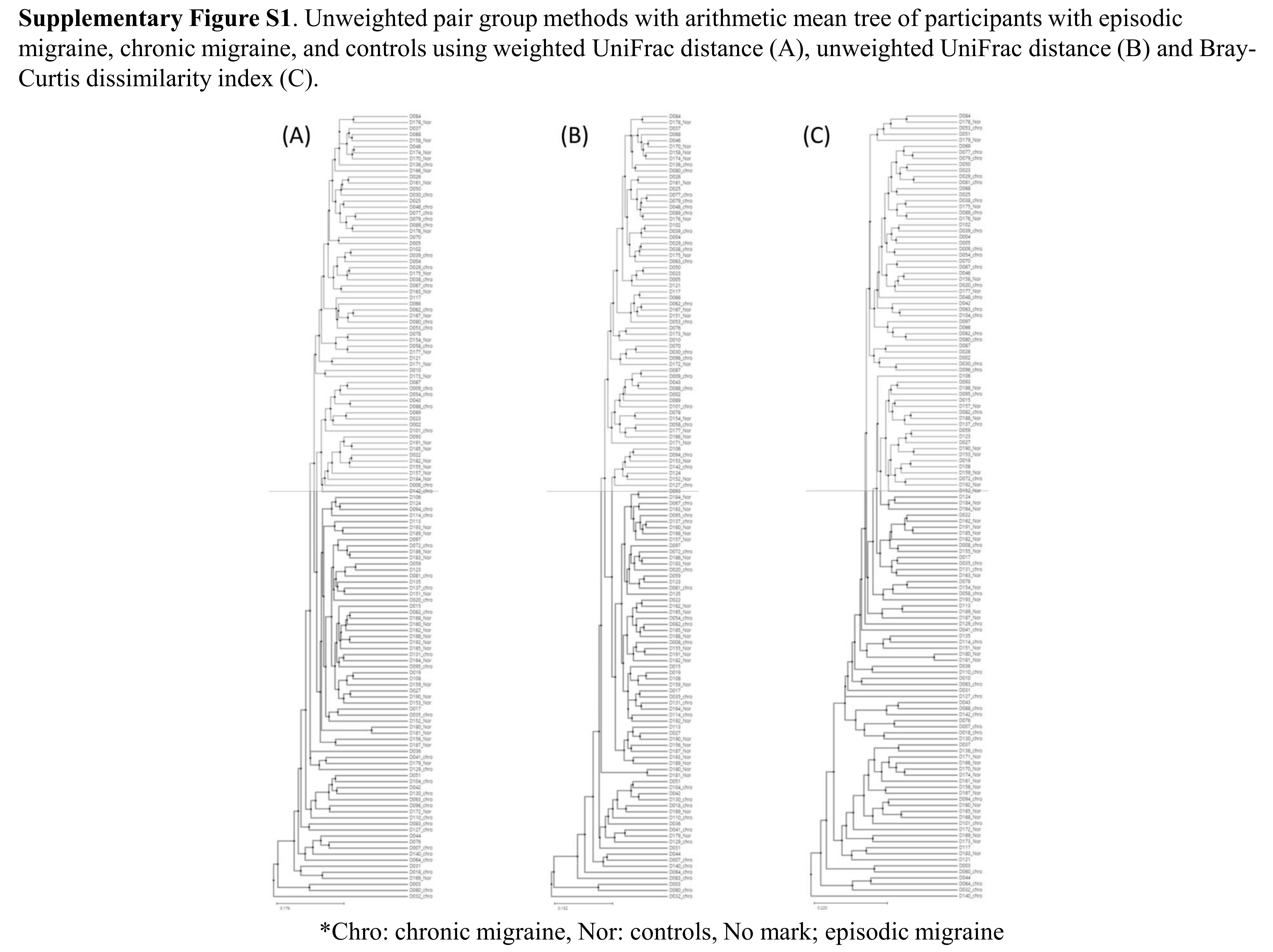

Supplement: Supplementary file 1 — Supplementary Information 1. [file 41598_2023_27586_MOESM1_ESM.jpeg]

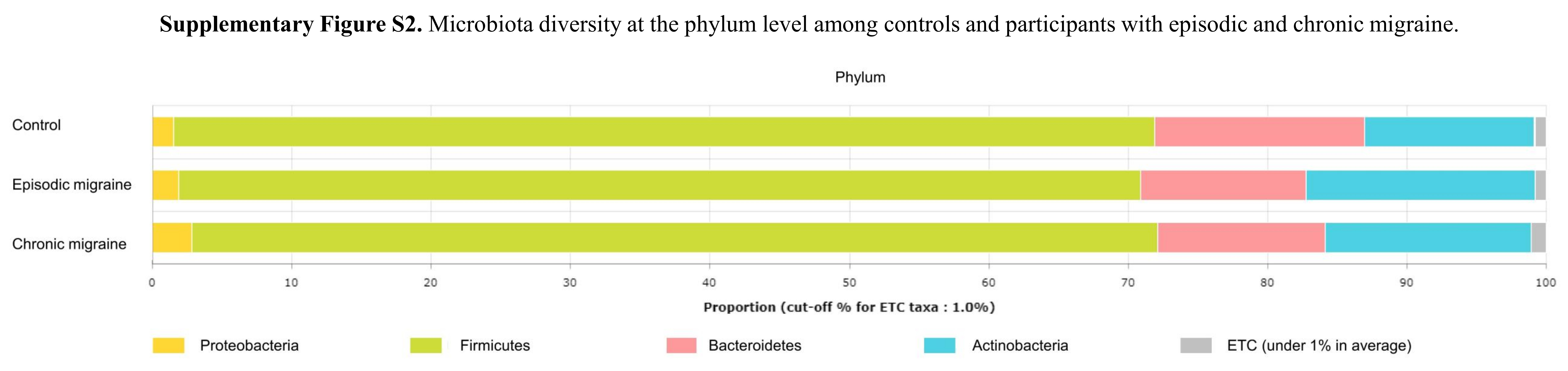

Supplement: Supplementary file 2 — Supplementary Information 2. [file 41598_2023_27586_MOESM2_ESM.jpeg]

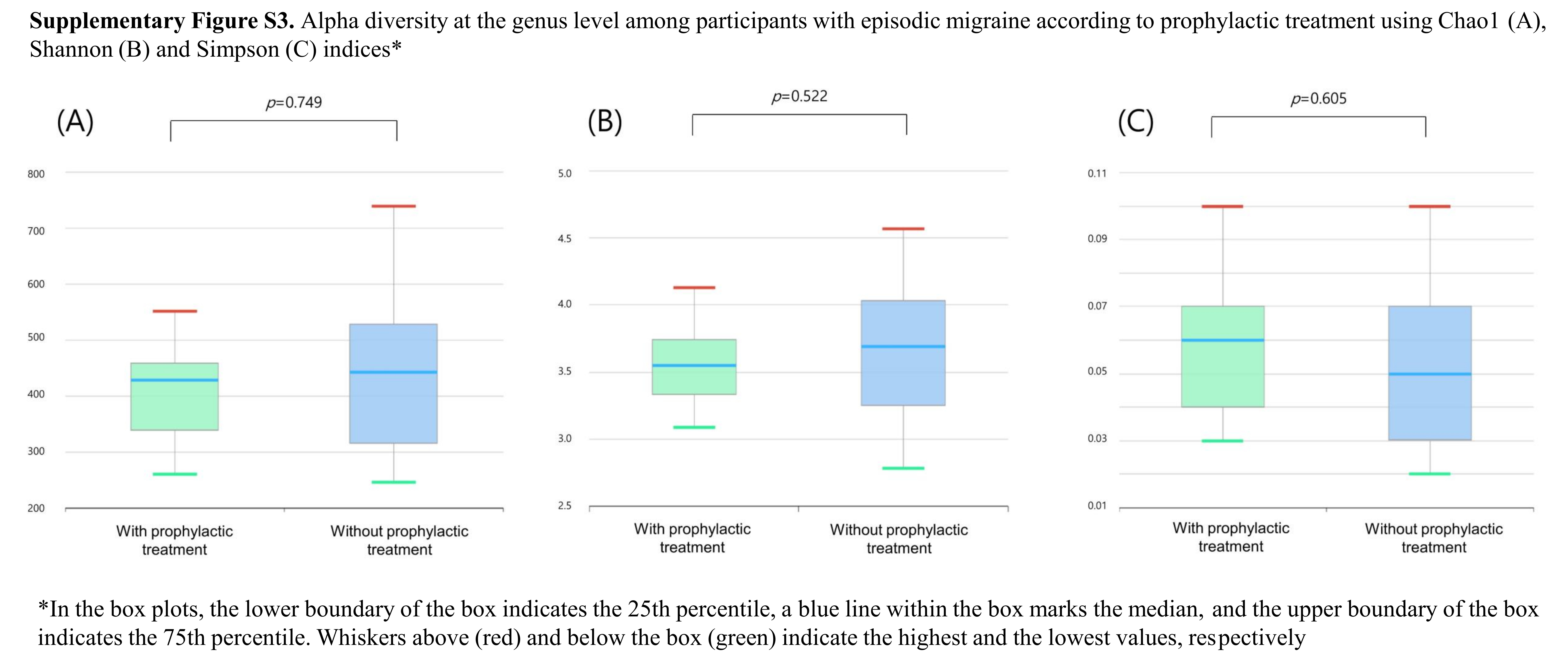

Supplement: Supplementary file 3 — Supplementary Information 3. [file 41598_2023_27586_MOESM3_ESM.jpeg]

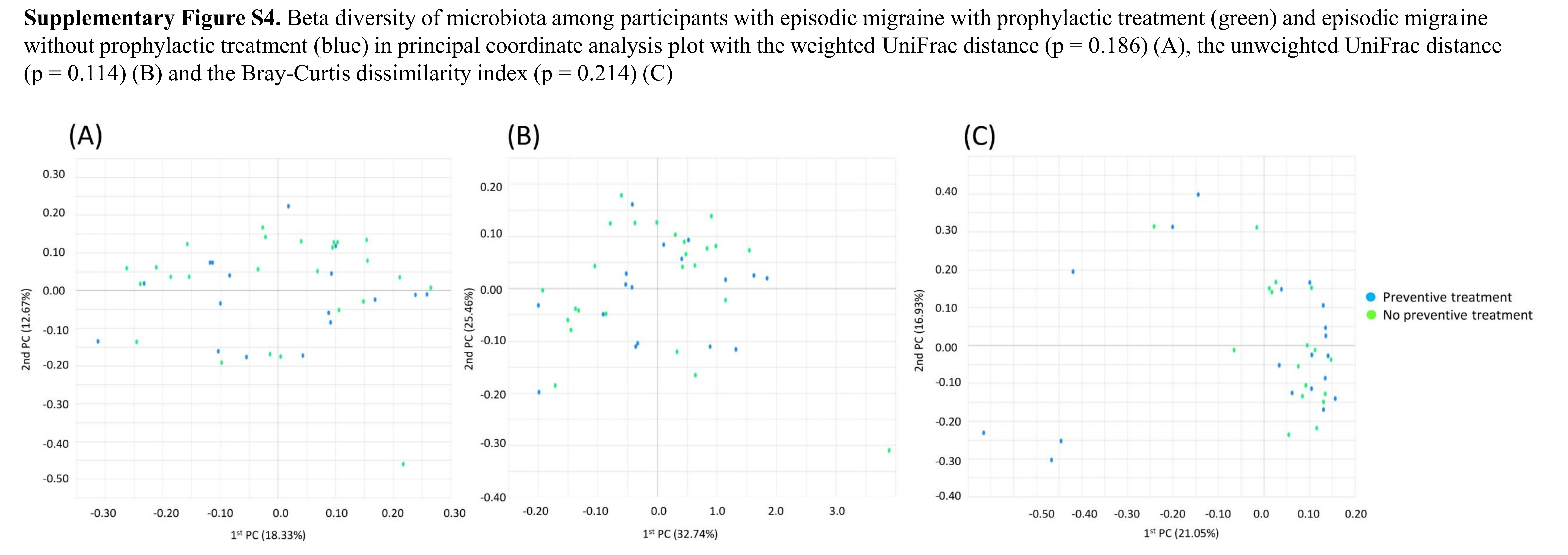

Supplement: Supplementary file 4 — Supplementary Information 4. [file 41598_2023_27586_MOESM4_ESM.jpeg]

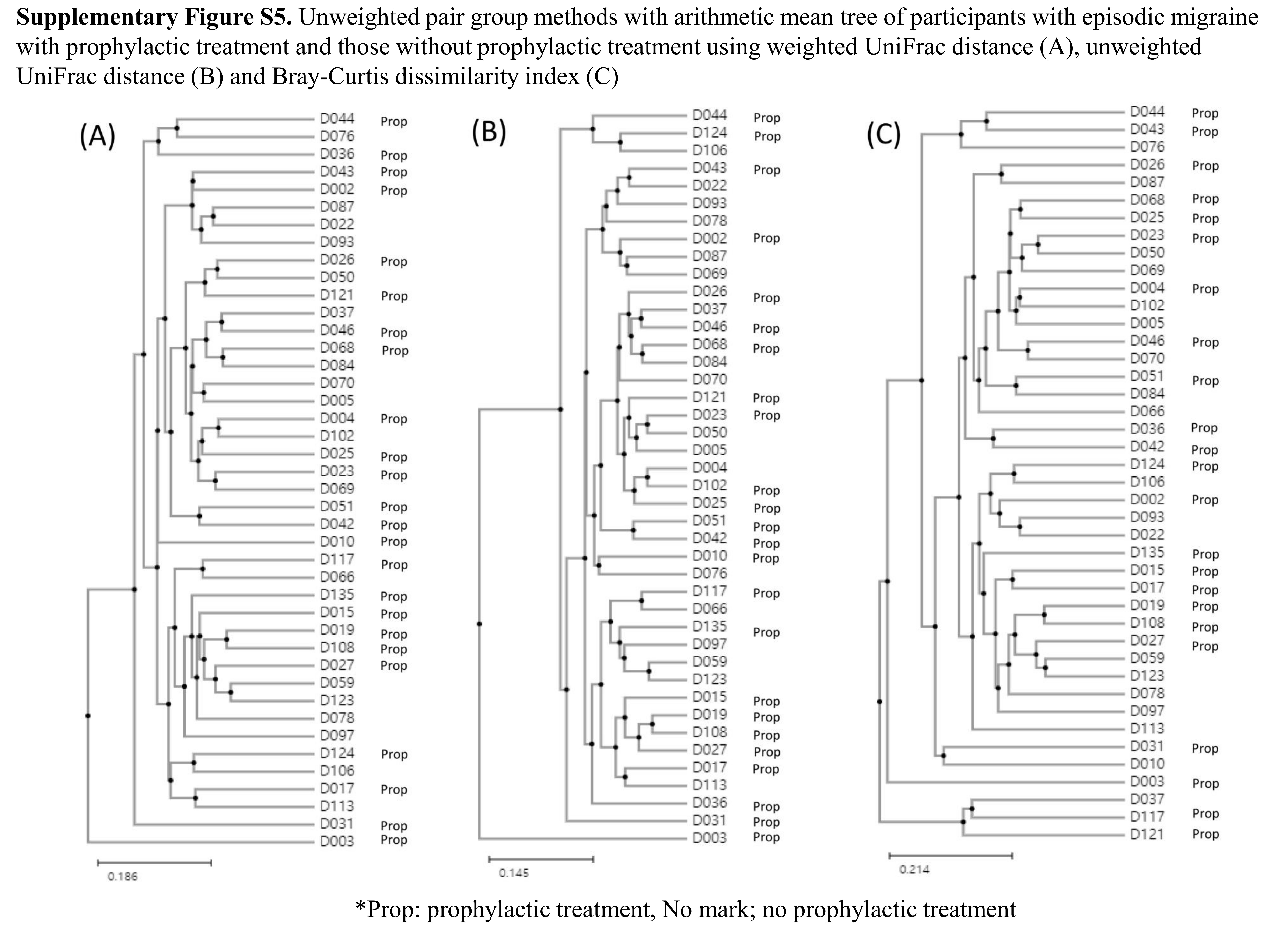

Supplement: Supplementary file 5 — Supplementary Information 5. [file 41598_2023_27586_MOESM5_ESM.jpeg]

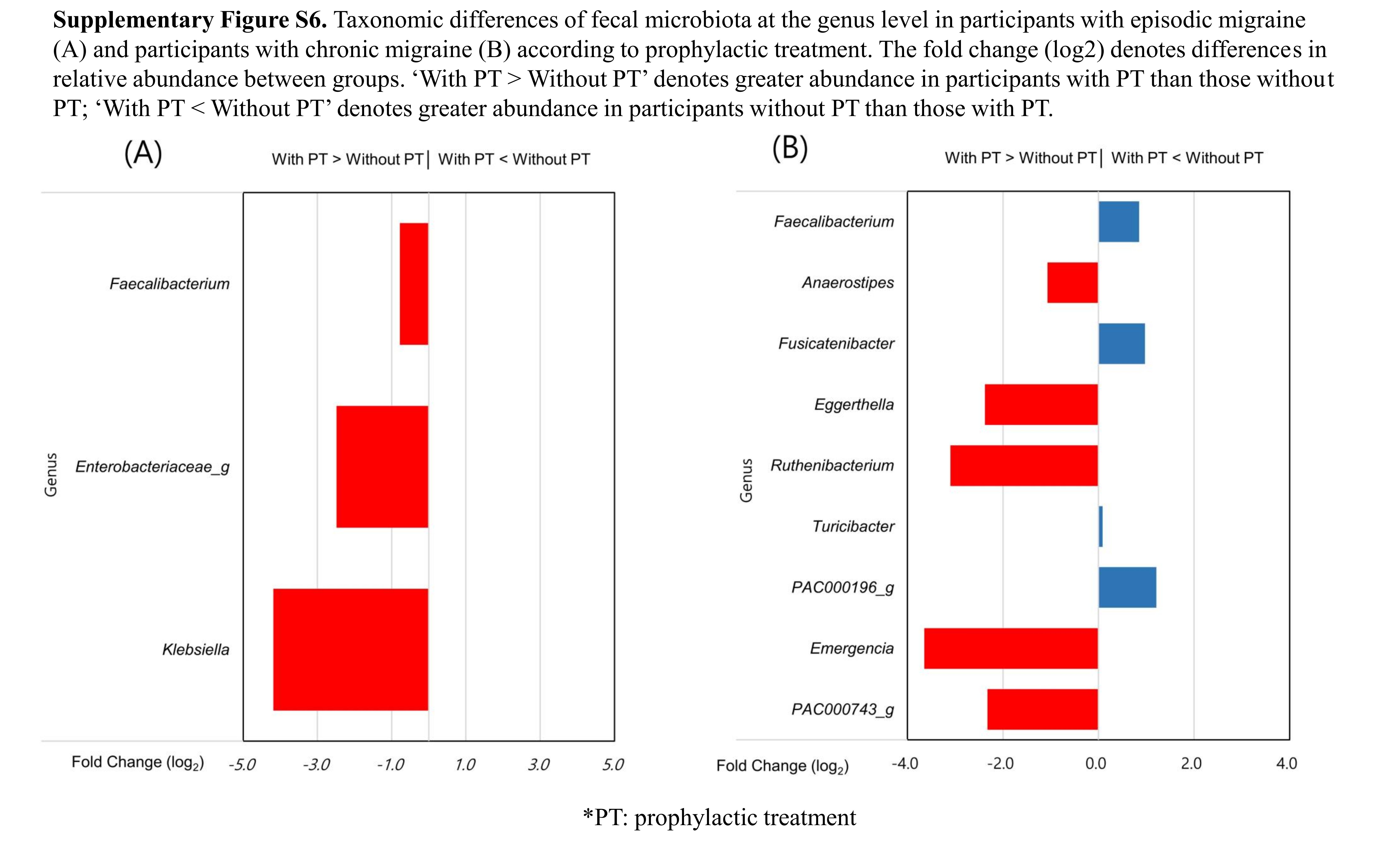

Supplement: Supplementary file 6 — Supplementary Information 6. [file 41598_2023_27586_MOESM6_ESM.jpeg]

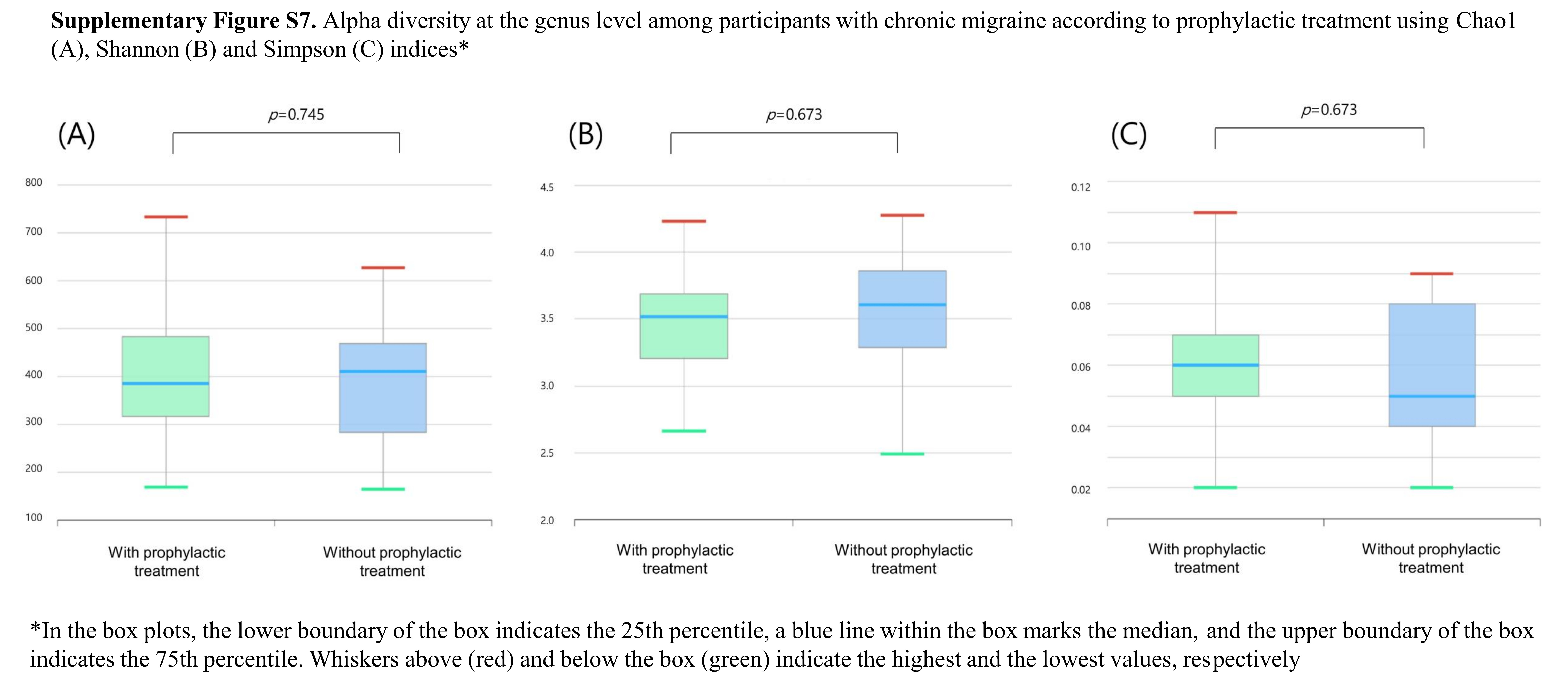

Supplement: Supplementary file 7 — Supplementary Information 7. [file 41598_2023_27586_MOESM7_ESM.jpeg]

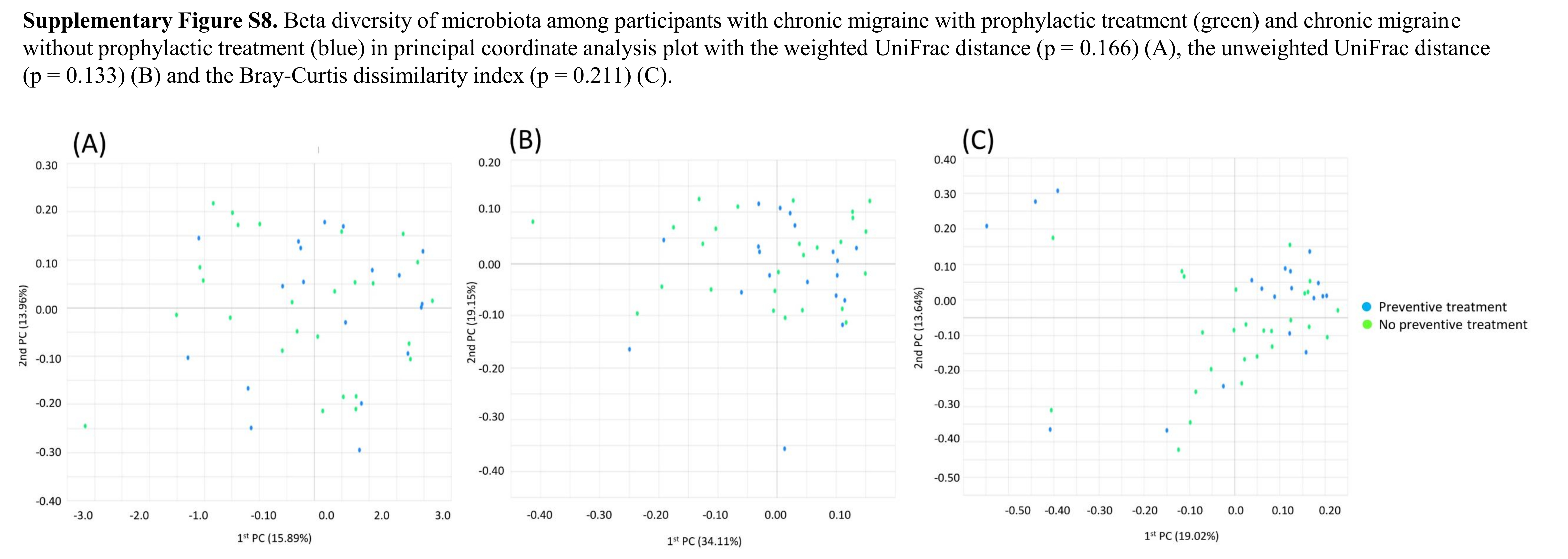

Supplement: Supplementary file 8 — Supplementary Information 8. [file 41598_2023_27586_MOESM8_ESM.jpeg]

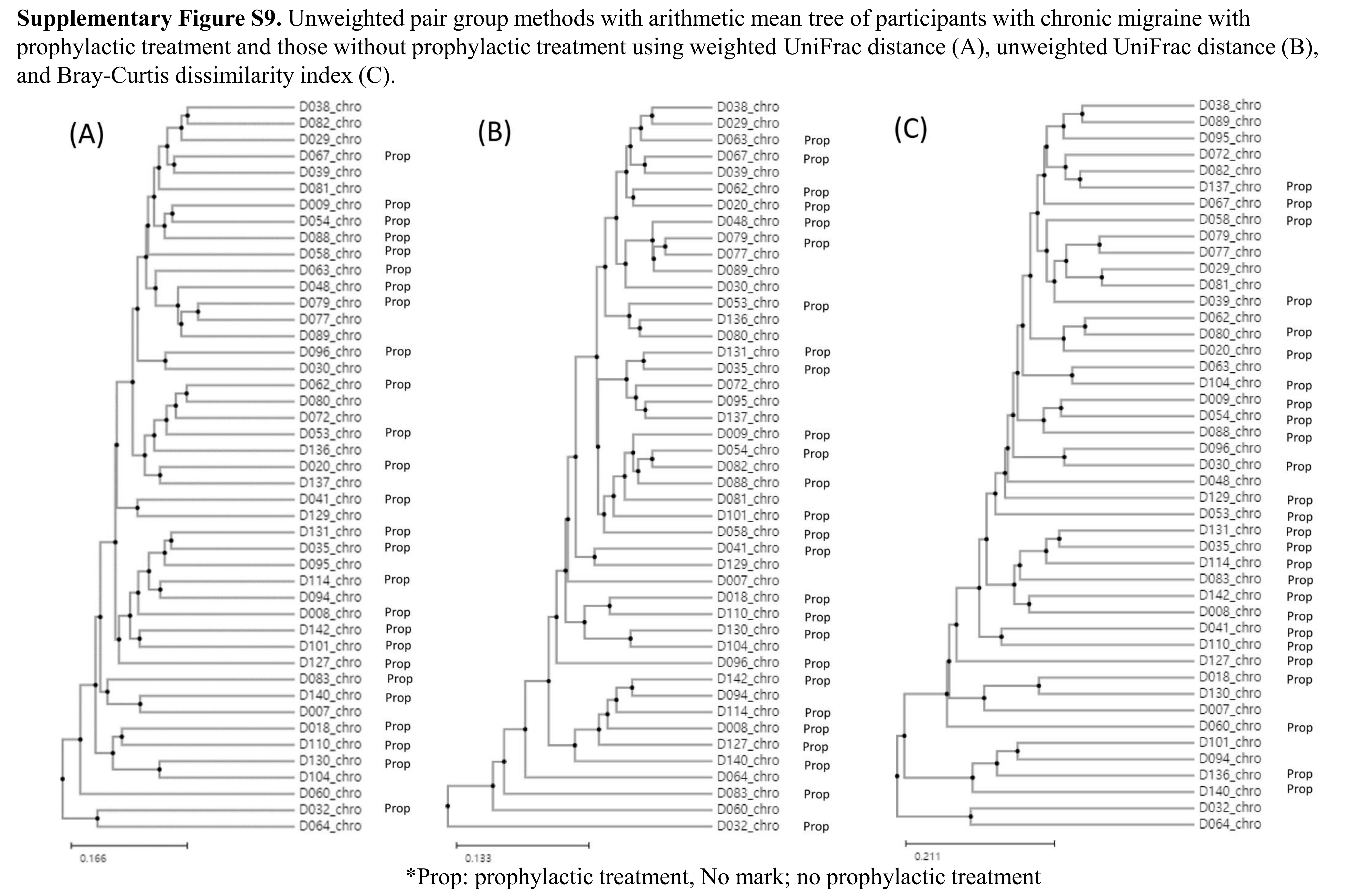

Supplement: Supplementary file 9 — Supplementary Information 9. [file 41598_2023_27586_MOESM9_ESM.jpeg]
