# Supplementary material for: Altered gut microbiota in individuals with episodic and chronic migraine
Source: Sci Rep. 2023 Jan 12;13:626. doi: 10.1038/s41598-023-27586-4 (PMC9835027; doi:10.1038/s41598-023-27586-4)
Supplement: Supplementary file 10 — Supplementary Information. [file 41598_2023_27586_MOESM10_ESM.docx]

**Supplementary files**

**File names:** Supplementary Figure S1A, B & C; Supplementary Figure S2; Supplementary Figure S3A, B & C; Supplementary Figure S4A, B & C; Supplementary Figure S5A, B & C; Supplementary Figure S6A & B; Supplementary Figure S7A & B; Supplementary Figure S8A & B; and Supplementary Figure S9A & B.

**File format:** jpeg

Title of data:

**Supplementary Figure S1.** Unweighted pair group methods with arithmetic mean tree of participants with episodic migraine, chronic migraine, and controls using weighted UniFrac distance (A), unweighted UniFrac distance (B) and Bray-Curtis dissimilarity index (C).

*Chro: chronic migraine, Nor: controls, No mark; episodic migraine

**Supplementary Figure S2.** Microbiota diversity at the phylum level among controls and participants with episodic and chronic migraine.

**Supplementary Figure S3**. Alpha diversity at the genus level among participants with episodic migraine according to prophylactic treatment using Chao1 (A), Shannon (B) and Simpson (C) indices*

*In the box plots, the lower boundary of the box indicates the 25th percentile, a blue line within the box marks the median, and the upper boundary of the box indicates the 75th percentile. Whiskers above (red) and below the box (green) indicate the highest and the lowest values, respectively

**Supplementary Figure S4.** Beta diversity of microbiota among participants with episodic migraine with prophylactic treatment (green) and episodic migraine without prophylactic treatment (blue) in principal coordinate analysis plot with the weighted UniFrac distance (*p* = 0.186) (A), the unweighted UniFrac distance (*p* = 0.114) (B) and the Bray-Curtis dissimilarity index (*p* = 0.214) (C)

**Supplementary Figure S5.** Unweighted pair group methods with arithmetic mean tree of participants with episodic migraine with prophylactic treatment and those without prophylactic treatment using weighted UniFrac distance (A), unweighted UniFrac distance (B) and Bray-Curtis dissimilarity index (C)

*Prop: prophylactic treatment, No mark; no prophylactic treatment

**Supplementary Figure S6.** Taxonomic differences of fecal microbiota at the genus level in participants with episodic migraine (A) and participants with chronic migraine (B) according to prophylactic treatment. The fold change (log_2_) denotes differences in relative abundance between groups. ‘With PT > Without PT’ denotes greater abundance in participants with PT than those without PT; ‘With PT < Without PT’ denotes greater abundance in participants without PT than those with PT

PT: prophylactic treatment

**Supplementary Figure S7.** Alpha diversity at the genus level among participants with chronic migraine according to prophylactic treatment using Chao1 (A), Shannon (B) and Simpson (C) indices*

*In the box plots, the lower boundary of the box indicates the 25th percentile, a blue line within the box marks the median, and the upper boundary of the box indicates the 75th percentile. Whiskers above (red) and below the box (green) indicate the highest and the lowest values, respectively

**Supplementary Figure S8.** Beta diversity of microbiota among participants with chronic migraine with prophylactic treatment (green) and chronic migraine without prophylactic treatment (blue) in principal coordinate analysis plot with the weighted UniFrac distance (*p* = 0.166) (A), the unweighted UniFrac distance (*p* = 0.133) (B) and the Bray-Curtis dissimilarity index (*p* = 0.211) (C).

**Supplementary Figure S9.** Unweighted pair group methods with arithmetic mean tree of participants with chronic migraine with prophylactic treatment and those without prophylactic treatment using weighted UniFrac distance (A), unweighted UniFrac distance (B), and Bray-Curtis dissimilarity index (C).

*Prop: prophylactic treatment, No mark; no prophylactic treatment
